# Supplementary material for: Levofloxacin and Ciprofloxacin Co-Crystals with Flavonoids: Solid-State Investigation for a Multitarget Strategy against Helicobacter pylori
Source: Pharmaceutics. 2024 Jan 30;16(2):203. doi: 10.3390/pharmaceutics16020203 (PMC10892363; doi:10.3390/pharmaceutics16020203)
Supplement: Supplementary file 1 [file pharmaceutics-16-00203-s001.zip › pharmaceutics-2849487-supplementary.pdf]

Supporting information (10 pages)

Levofloxacin and Ciprofloxacin Co-Crystals with Flavonoids: Solid-State Investigation for a Multitarget Strategy against *Helicobacter pylori*

Cecilia Fiore [1,2,*], Federico Antoniciello [3,*], Davide Roncarati [3], Vincenzo Scarlato [3], Fabrizia Grepioni [1] and Dario Braga [1]

[1] Department of Chemistry "Giacomo Ciamician", University of Bologna, Via Selmi 2, 40126 Bologna, Italy; fabrizia.grepioni@unibo.it (F.G.); dario.braga@unibo.it (D.B.)
[2] Department of Applied Science and Technology (DISAT), Politecnico di Torino, Corso Duca degli Abruzzi 24, 10129 Torino, Italy
[3] Department of Pharmacy and Biotechnology (FaBiT), University of Bologna, Via Selmi 3, 40126 Bologna, Italy; davide.roncarati@unibo.it (D.R.); vincenzo.scarlato@unibo.it (V.S.)
* Correspondence: cecilia.fiore@polito.it (C.F.); federic.antoniciell2@unibo.it (F.A.); Tel.: +39-051-2094207 (F.A.)

Powder X-ray Diffraction (PXRD) ....page 2

Thermogravimetric analysis (TGA) ....page 4

^{1}H NMR spectroscopy ....page 5

Powder X-ray Diffraction (PXRD)

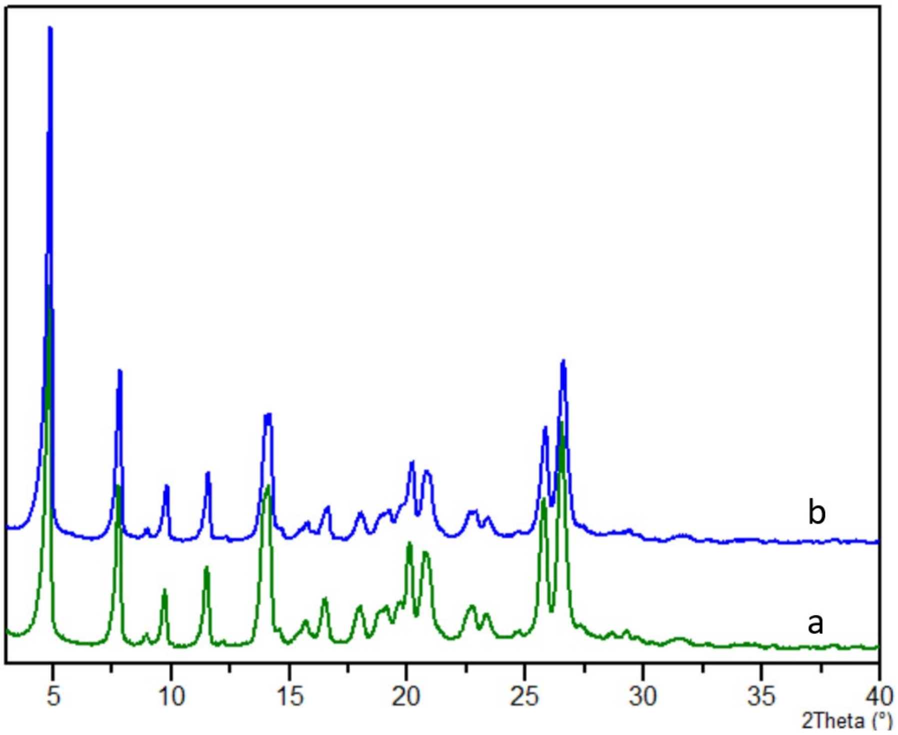

Figure S1 Comparison of the experimental PXRD patterns for LEV·QUE·xEtOH as obtained from ball milling (a, green) and slurry (b, blue).

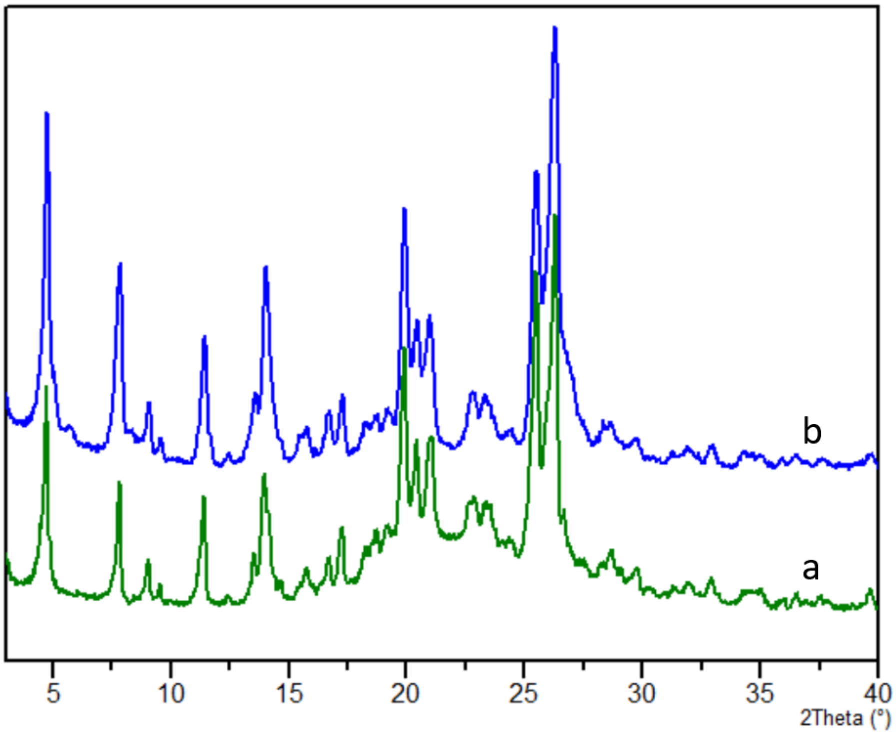

Figure S2 Comparison of the experimental PXRD patterns for LEV·MYR·xEtOH as obtained from ball milling (a, green) and slurry (b, blue).

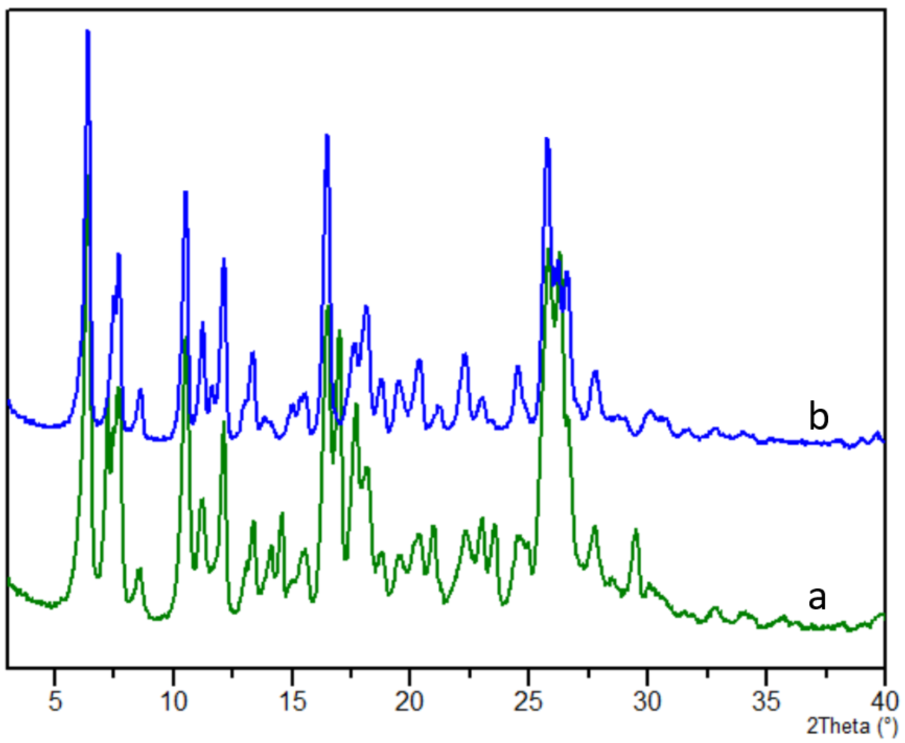

Figure S3 Comparison of the experimental PXRD patterns for LEV_2·HES as obtained from ball milling (a, green) and slurry (b, blue). (The ball milling product still contains unreacted substance).

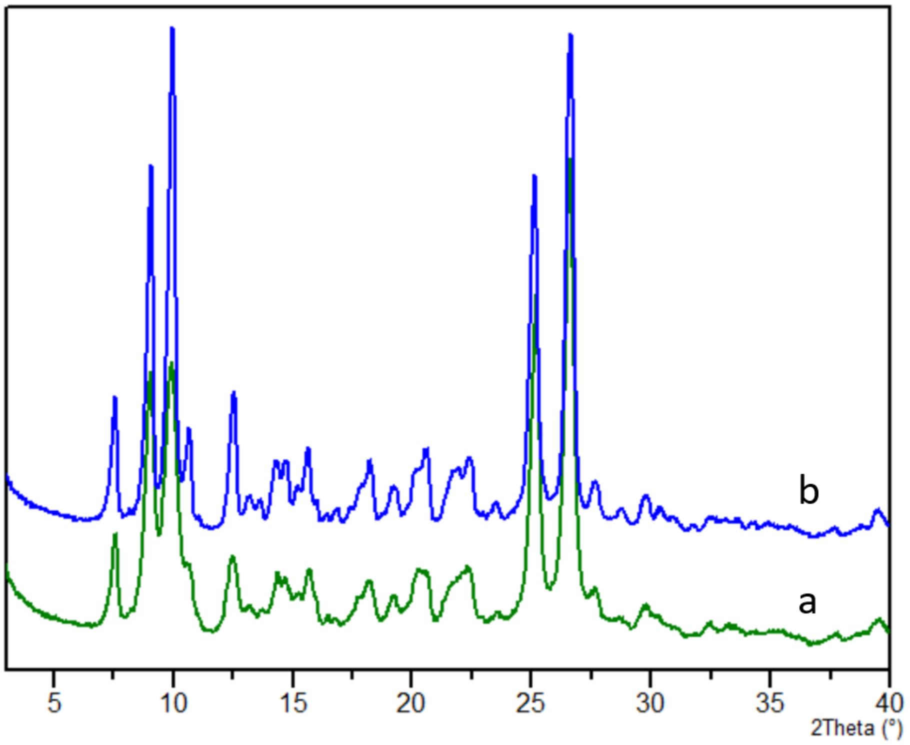

Figure S4 Comparison of the experimental PXRD patterns for CIP·QUE as obtained from ball milling (a, green) and slurry (b, blue).

Thermogravimetric Analysis (TGA)

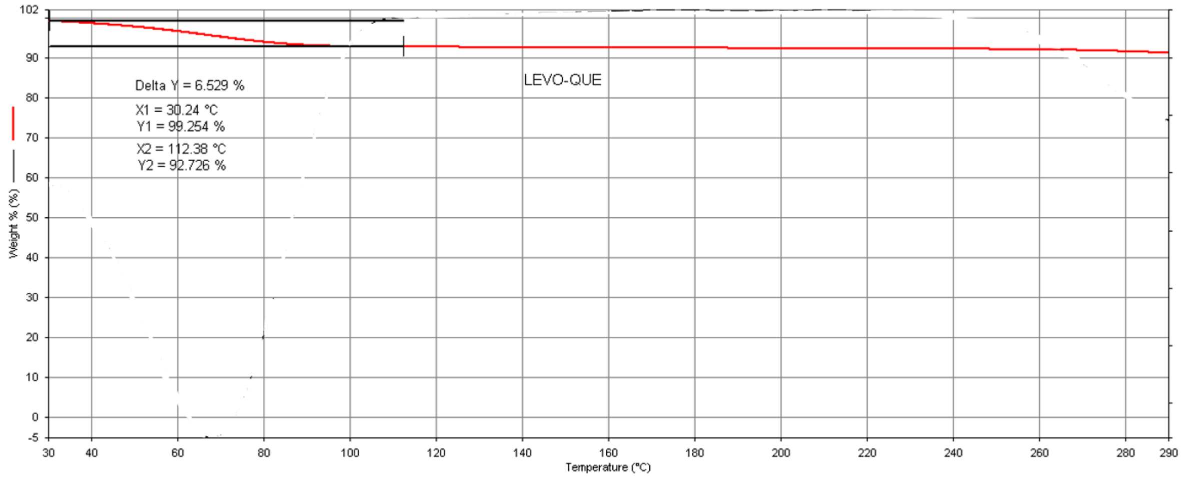

Figure S5. TGA trace for LEV·QUE·xEtOH.

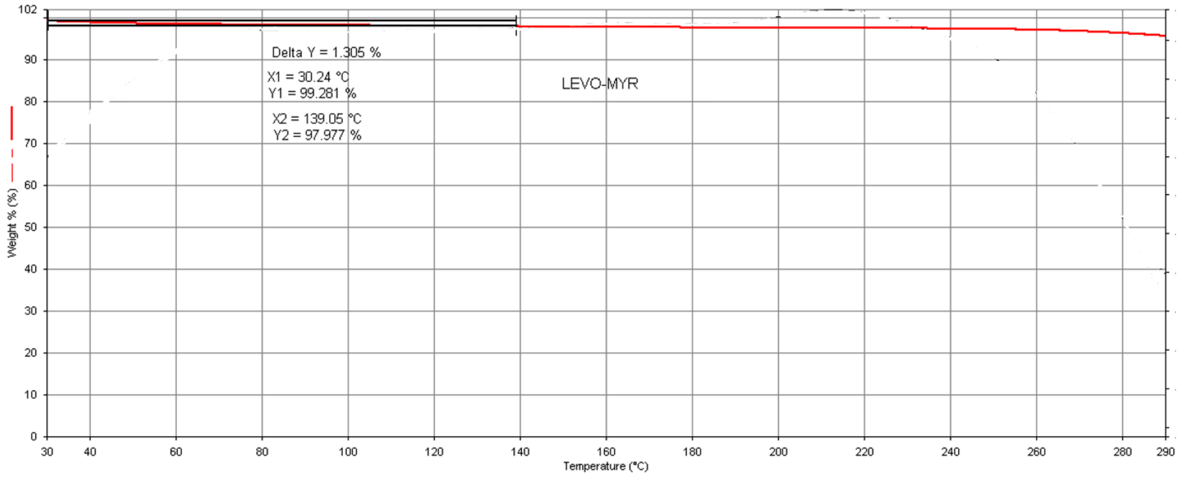

Figure S6. TGA trace for LEV·MYR·xEtOH.

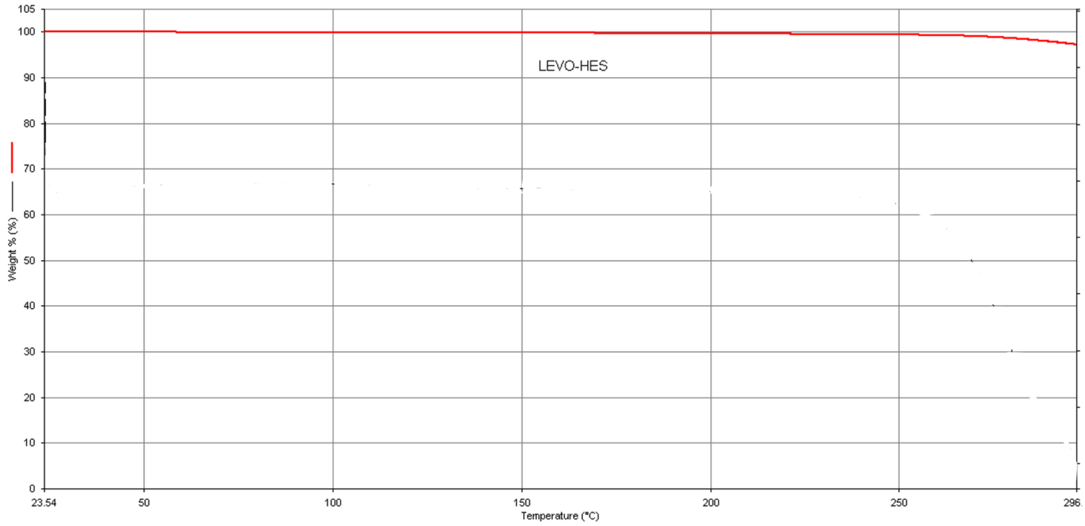

Figure S7. TGA trace for LEV_2·HES

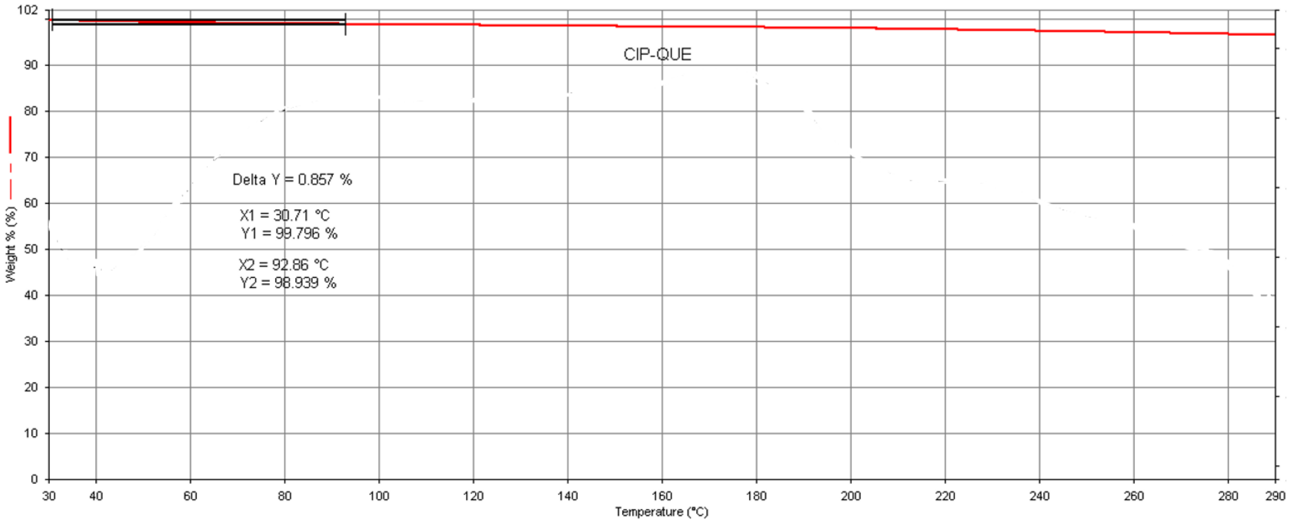

Figure S8. TGA trace for CIP·QUE.

^{1}H NMR spectroscopy

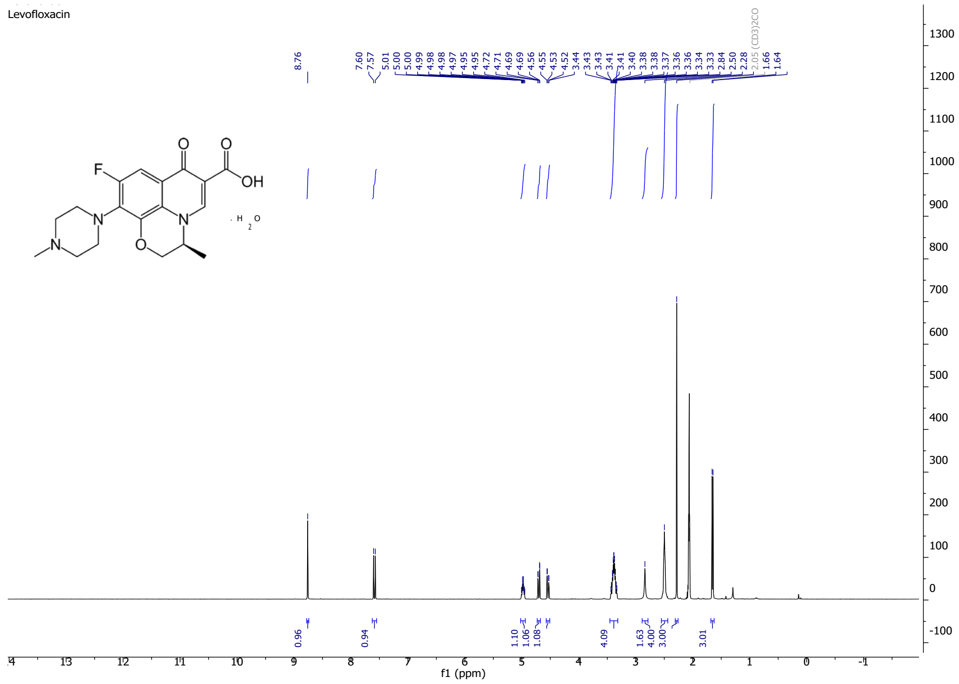

Figure S9. Levofloxacin: ^{1}H NMR (401 MHz, Acetone-d_6) δ 8.76 (s, 1H), 7.59 (d, *J* = 12.4 Hz, 1H), 5.02 – 4.94 (m, 1H), 4.70 (dd, *J* = 11.5, 1.8 Hz, 1H), 4.54 (dd, *J* = 11.5, 2.3 Hz, 1H), 3.39 (m , 4H), 2.50 (s, 4H), 2.28 (s, 3H), 1.65 (d, *J* = 6.8 Hz, 3H). **H_2O:** 2.84 (s, 2H).

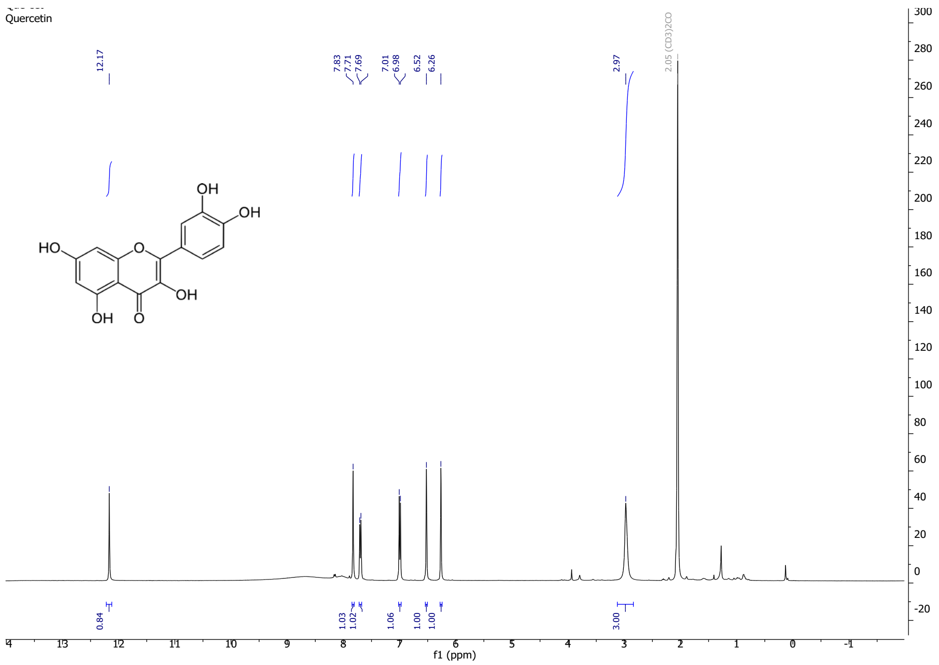

Figure S10. Quercetin: ^{1}H NMR (401 MHz, Acetone-d_6) δ 12.17 (s, 1H), 7.83 (s, 1H), 7.70 (d, *J* = 8.4 Hz, 1H), 7.00 (d, *J* = 8.5 Hz, 1H), 6.52 (s, 1H), 6.26 (s, 1H), 2.97 (s, 3H).

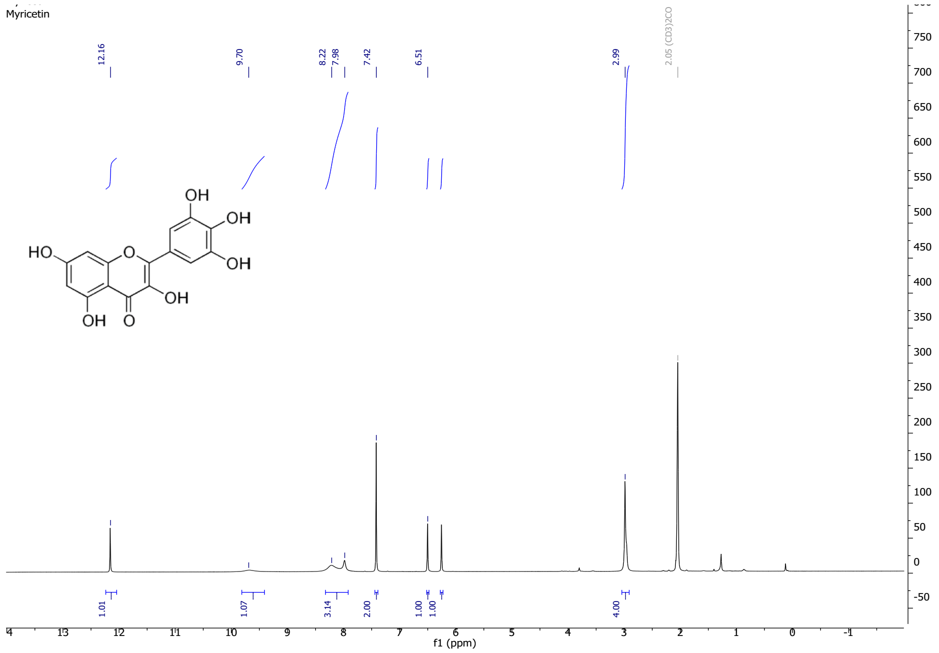

Figure S11. Myricetin: ^{1}H NMR (401 MHz, Acetone-d_6) δ 12.16 (s, 1H), 9.70 (s, 1H), 8.10 (d, *J* = 93.4 Hz, 3H), 7.42 (s, 2H), 6.51 (s, 1H), 6.26 (d, *J* = 2.1 Hz, 1H), 2.99 (s, 4H – MYRICETIN + H_2O/SOLVENT).

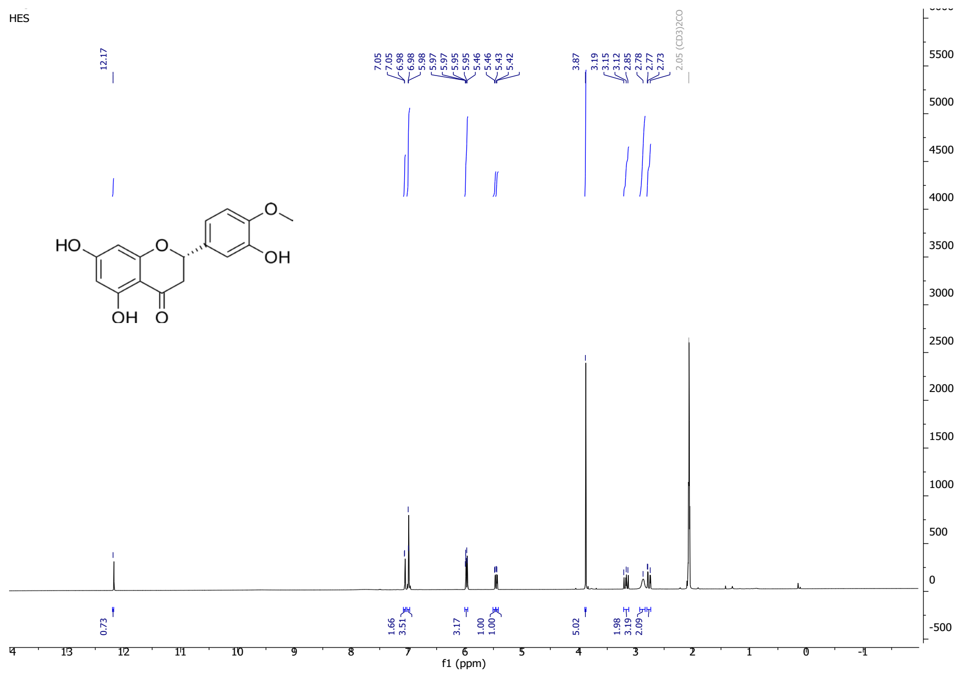

Figure S12. Hesperetin: ^{1}H NMR (401 MHz, Acetone-d_6) δ 12.17 (s, 1H), 7.05 (d, *J* = 1.4 Hz, 2H), 6.98 (d, *J* = 2.0 Hz, 4H), 5.99 – 5.94 (m, 3H), 5.46 (d, *J* = 3.1 Hz, 1H), 5.43 (d, *J* = 3.1 Hz, 1H). -O-CH_3 + solvent impurity :3.87 (s, 5H), SOLVENTS RESIDUES 3.20 – 3.11 (m, 2H), 2.85 (s, 3H), 2.79 – 2.72 (m, 2H).

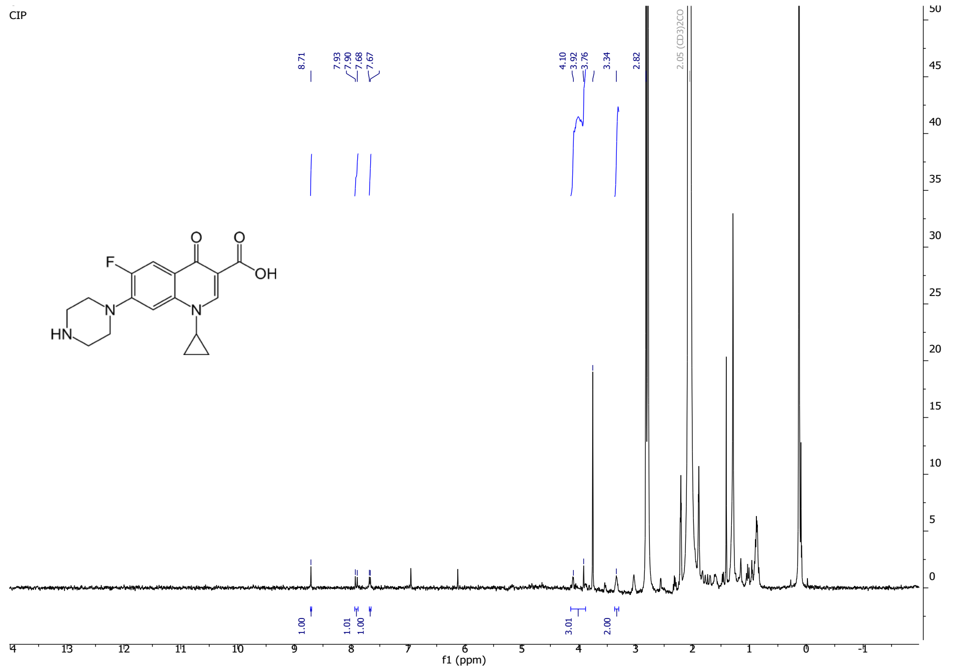

Figure S13. Ciprofloxacin: analysis of the spectrum incomplete due to the low quality of signals belonging to CIP and too the presence of impurities covering the signals of interest. ^{1}H NMR (401 MHz, Acetone-d_6) δ 8.71 (s, 1H), 7.91 (d, *J* = 13.6 Hz, 1H), 7.68 (d, *J* = 7.0 Hz, 1H), 4.01 (d, *J* = 72.0 Hz, 3H), 3.34 (s, 2H).

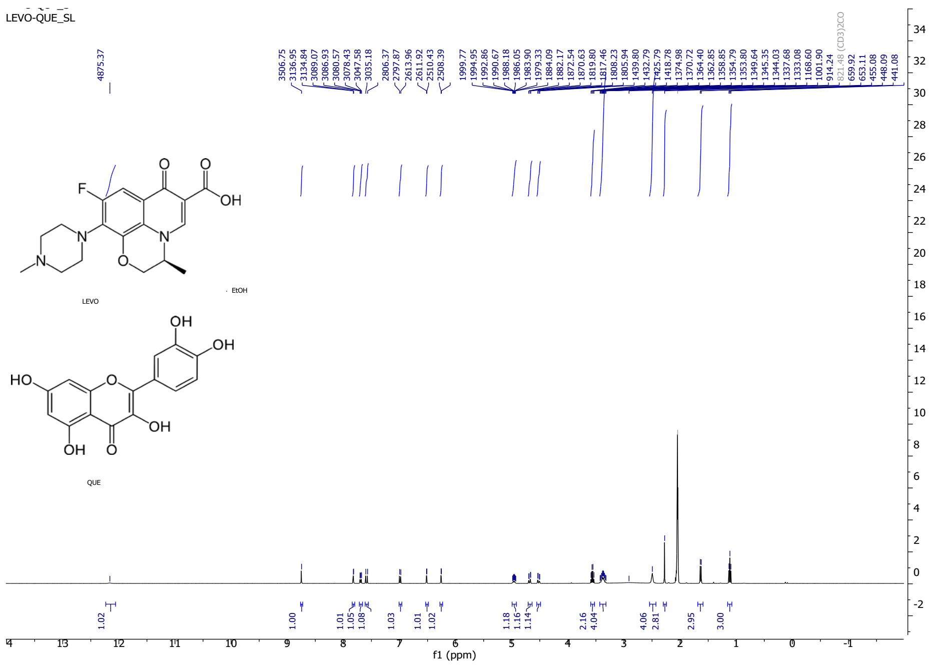

Figure S14. LEV·QUE·xEtOH: ^{1}H NMR (401 MHz, Acetone-d_6) δ 12.17 (s, 1H), 8.75 (s, 1H), 7.83 (d, *J* = 2.1 Hz, 1H), 7.70 (dd, *J* = 8.5, 2.1 Hz, 1H), 7.59 (d, *J* = 12.4 Hz, 1H), 6.99 (d, *J* = 8.5 Hz, 1H), 6.52 (d, *J* = 2.0 Hz, 1H), 6.26 (d, *J* = 2.0 Hz, 1H), 4.96 (m, 1H), 4.68 (dd, *J* = 11.5, 1.9 Hz, 1H), 4.52 (dd, *J* = 11.5, 2.3 Hz, 1H), 3.57 (q, *J* = 7.0 Hz, 2H), 3.44 – 3.33 (m, 4H), 2.50 (s, 4H), 2.28 (s, 3H), 1.64 (d, *J* = 6.8 Hz, 3H), **EtOH -CH_2** :3.57 (q, *J* = 7.0 Hz, 2H), **-CH_3**: 1.12 (t, *J* = 7.0 Hz, 3H).

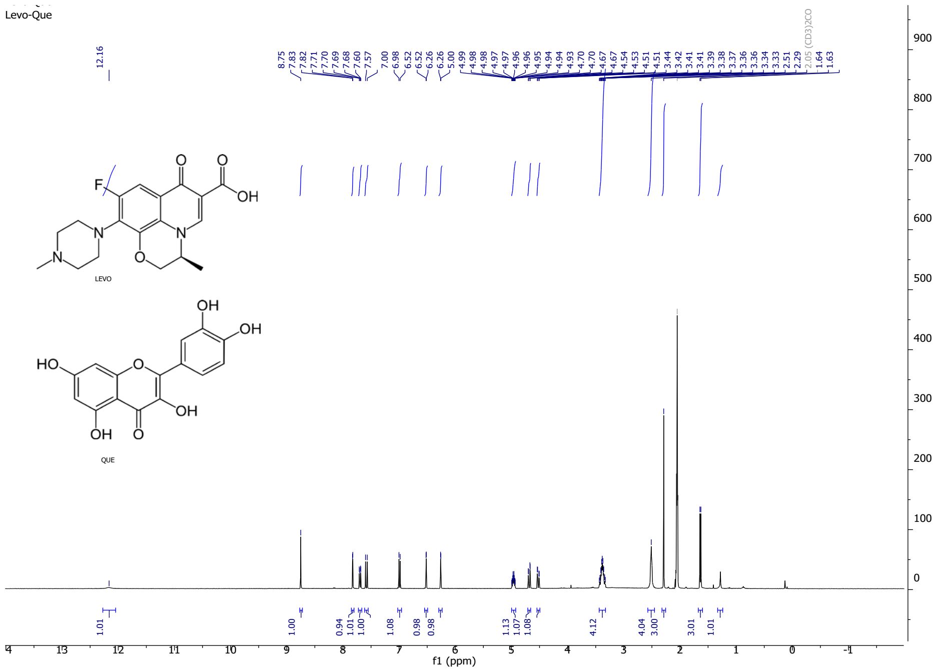

Figure S15. LEV·QUE: ^{1}H NMR (401 MHz, Acetone-d_6) δ 12.16 (s, 1H), 8.75 (s, 1H), 7.83 (d, *J* = 2.1 Hz, 1H), 7.70 (dd, *J* = 8.5, 2.1 Hz, 1H), 7.58 (d, *J* = 12.4 Hz, 1H), 6.99 (d, *J* = 8.5 Hz, 1H), 6.52 (d, *J* = 1.9 Hz, 1H), 6.26 (d, *J* = 2.0 Hz, 1H), 4.96 (m, 1H), 4.68 (dd, *J* = 11.5, 1.8 Hz, 1H), 4.52 (dd, *J* = 11.5, 2.3 Hz, 1H), 3.44 – 3.33 (m, 4H), 2.51 (s, 4H), 2.29 (s, 3H), 1.64 (d, *J* = 6.8 Hz, 3H), 1.28 (s, 1H).

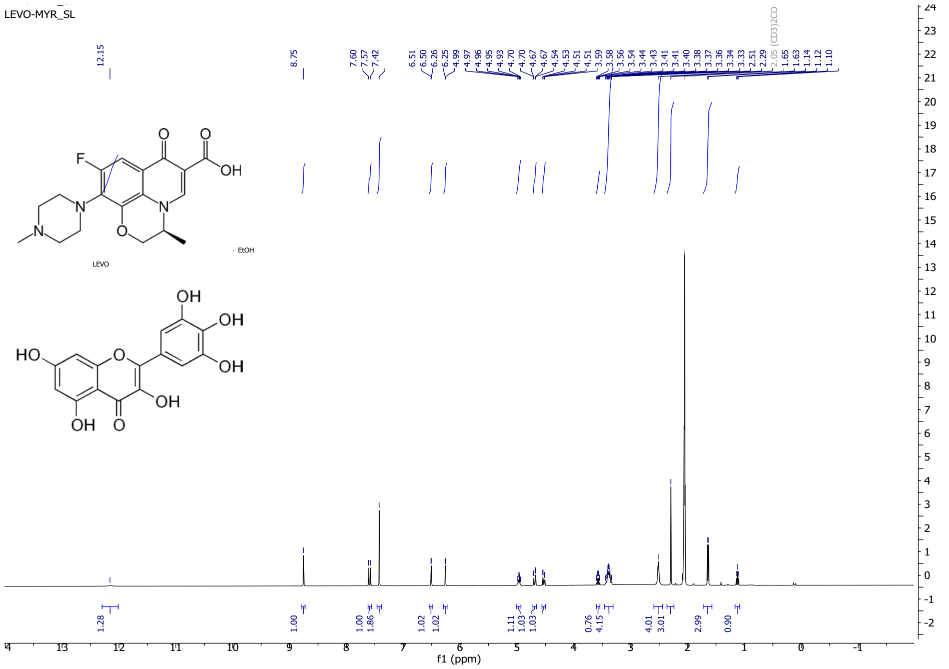

Figure S16. LEV·MYR·xEtOH: ^{1}H NMR (401 MHz, Acetone-d_6) δ 12.15 (s, 1H), 8.75 (s, 1H), 7.59 (d, J = 12.4 Hz, 1H), 7.42 (s, 2H), 6.50 (d, J = 2.0 Hz, 1H), 6.26 (d, J = 2.0 Hz, 1H), 5.01 – 4.92 (m, 1H), 4.68 (dd, J = 11.5, 1.9 Hz, 1H), 4.52 (dd, J = 11.5, 2.3 Hz, 1H), 3.38 (m, 4H), 2.51 (s, 4H), 2.29 (s, 3H), 1.64 (d, J = 6.8 Hz, 3H). **EtOH -CH_2** :3.57 (q, J = 7.0 Hz, 0.76H), **-CH_3** :1.12 (t, J = 7.0 Hz, 0.90H).

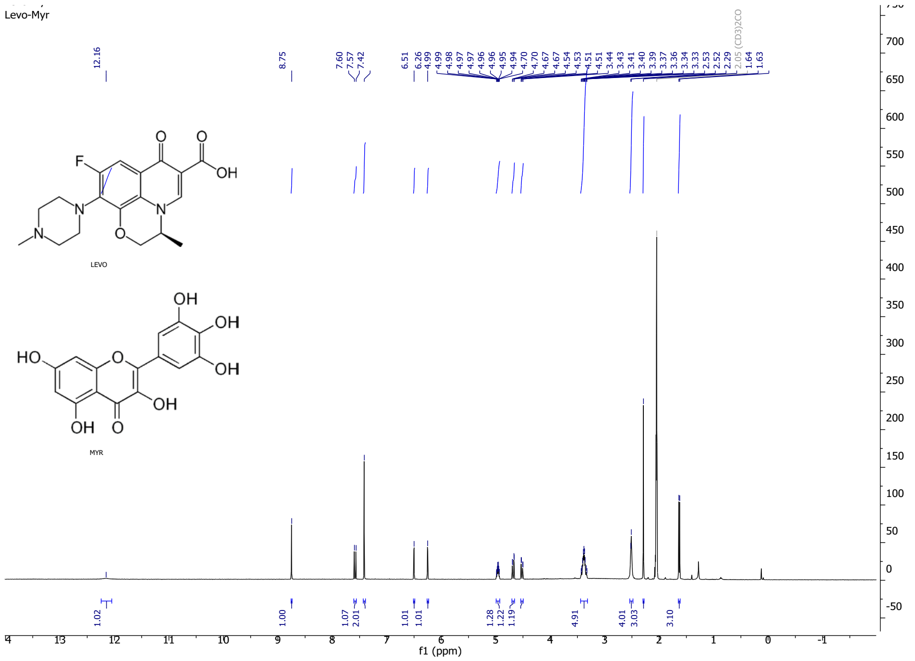

Figure S17. LEV·MYR: ^{1}H NMR (401 MHz, Acetone-d_6) δ 12.16 (s, 1H), 8.75 (s, 1H), 7.58 (d, J = 12.4 Hz, 1H), 7.42 (s, 2H), 6.51 (s, 1H), 6.26 (s, 1H), 5.00 – 4.93 (m, 1H), 4.68 (dd, J = 11.5, 1.8 Hz, 1H), 4.52 (dd, J = 11.6, 2.3 Hz, 1H), 3.45 – 3.32 (m, 5H), 2.55 – 2.49 (m, 4H), 2.29 (s, 3H), 1.64 (d, J = 6.8 Hz, 3H).

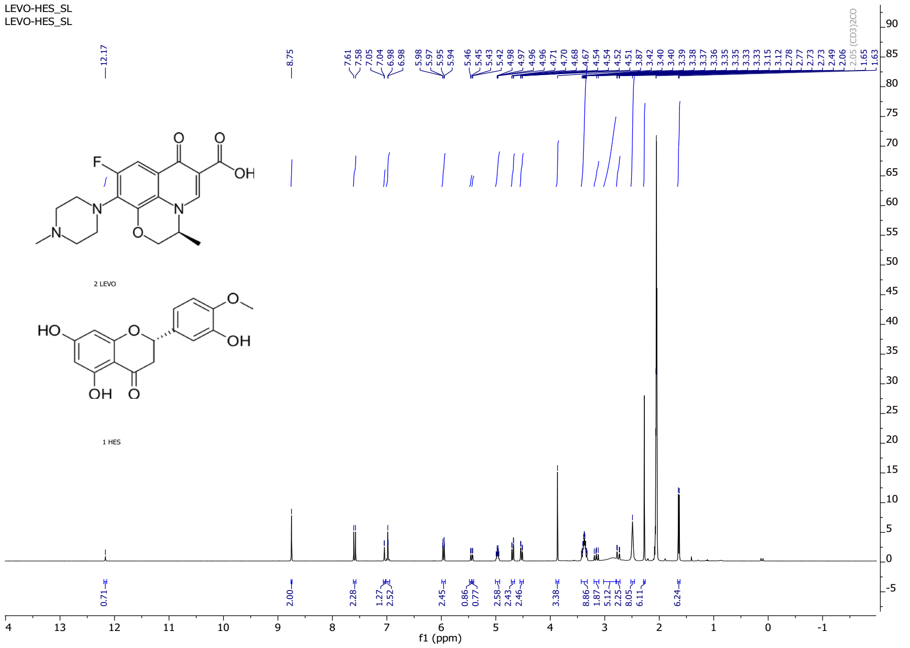

Figure S18. LEV$_2$·HES: ^{1}H NMR (401 MHz, Acetone-d_6) δ 12.17 (s, 1H), 8.75 (s, 2H), 7.59 (d, J = 12.4 Hz, 2H), 7.05 (d, J = 1.3 Hz, 1.5H), 6.98 (d, J = 2.1 Hz, 3H), 5.96 (dd, J = 10.5, 2.1 Hz, 2H), 5.46 (d, J = 3.0 Hz, 1H), 5.43 (d, J = 3.0 Hz, 1H), 4.97 (m, 3H), 4.69 (dd, J = 11.5, 1.9 Hz, 2H), 4.53 (dd, J = 11.5, 2.3 Hz, 2H), 3.87 (s, 3H), 3.38 (th, J = 12.1, 4.3 Hz, 8H), 3.16 (dd, J = 17.1, 12.6 Hz, 2H), 2.85 (s, 5H), 2.75 (dd, J = 17.1, 3.1 Hz, 2H), 2.49 (s, 8H), 2.27 (s, 6H), 1.64 (d, J = 6.8 Hz, 6H).

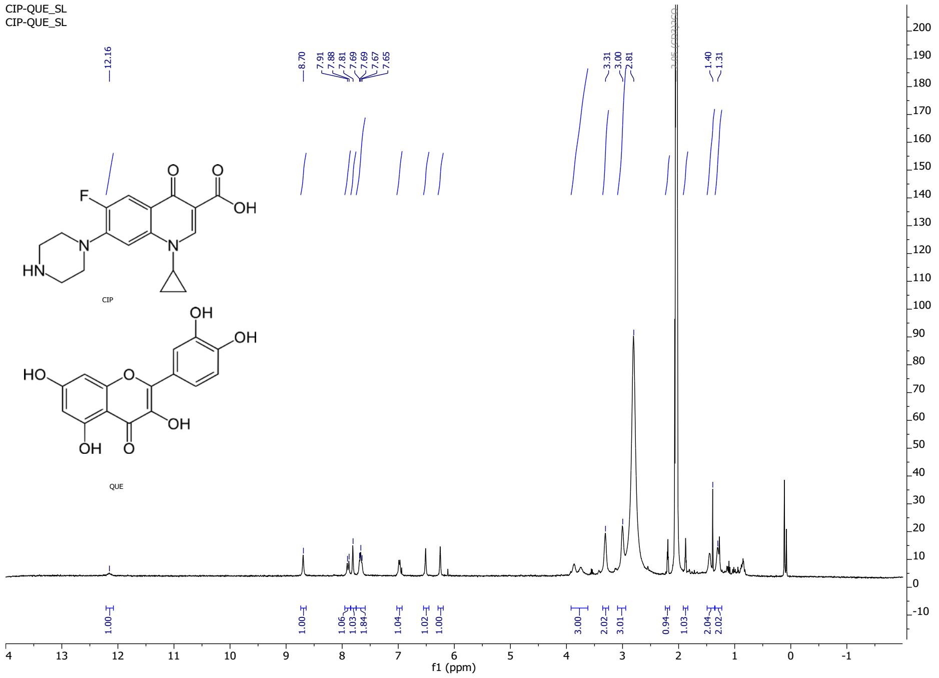

Figure S19. CIP·QUE: ^{1}H NMR (401 MHz, Acetone-d_6) δ 12.16 (s, 1H), 8.70 (s, 1H), 7.90 (d, J = 11.6 Hz, 1H), 7.81 (s, 1H), 7.76 – 7.59 (m, 2H), 7.03 – 6.94 (m, 1H), 6.52 (s, 1H), 6.25 (s, 1H), 3.81 (d, J = 49.0 Hz, 3H), 3.31 (s, 2H), 3.00 (s, 3H), 2.20 (dd, J = 4.5, 2.3 Hz, 1H), 1.88 (p, J = 2.2 Hz, 1H), 1.40 (s, 2H), 1.31 (s, 2H).
